# Cell Reprogramming, IPS Limitations, and Overcoming Strategies in Dental Bioengineering

**DOI:** 10.1155/2012/365932

**Published:** 2012-05-28

**Authors:** Gaskon Ibarretxe, Antonia Alvarez, Maria-Luz Cañavate, Enrique Hilario, Maitane Aurrekoetxea, Fernando Unda

**Affiliations:** Department of Cell Biology and Histology, Faculty of Medicine and Dentistry, University of the Basque Country (UPV/EHU), 48940 Leioa, Spain

## Abstract

The procurement of induced pluripotent stem cells, or IPS cells, from adult differentiated animal cells has the potential to revolutionize future medicine, where reprogrammed IPS cells may be used to repair disease-affected tissues on demand. The potential of IPS cell technology is tremendous, but it will be essential to improve the methodologies for IPS cell generation and to precisely evaluate each clone and subclone of IPS cells for their safety and efficacy. Additionally, the current state of knowledge on IPS cells advises that research on their regenerative properties is carried out in appropriate tissue and organ systems that permit a safe assessment of the long-term behavior of these reprogrammed cells. In the present paper, we discuss the mechanisms of cell reprogramming, current technical limitations of IPS cells for their use in human tissue engineering, and possibilities to overcome them in the particular case of dental regeneration.

## 1. Cell Dedifferentiation and Pluripotency

Cellular dedifferentiation underlies important issues in biology, such as tissue regeneration and cloning, and signifies the withdrawal of cells from a given differentiated state into a stem-cell-like state that confers pluripotency. Pluripotency *in vivo* pertains to the cells of early embryos that can generate all of the tissues in the organism. Embryonic stem cells (ESC) are preimplantation embryo-derived cells having three properties: self-renewal, pluripotency, and primary chimera formation [1]. ES cells represent invaluable tools for research into the mechanism of tissue formation. *In vitro* pluripotency may be maintained in ES cells, which are harvested from the inner cell mass of the blastocyst stage embryo. ES cells have demonstrated longevity in culture by maintaining their undifferentiated state for at least 80 passages. Moreover, if ES cells are cultured with the appropriate nutrients at their disposal, these cells can potentially give rise to all cell types of the body, including pluripotent germinal cells, and their offspring can become integrated in a tissue, adopting the character and behavior of the cells in this new tissue environment. However, there are also significant problems associated with the use of human ES cells.

- Their obtention involves manipulation of human embryos and therefore serious legal and ethical issues [2].
- If the transplanted cells differ genetically from the cells of the patient, the immune system of the latter may reject and destroy these cells and the patients would be on life-long immunosuppressants.
- Pluripotent stem cells (SCs) present a safety concern because of their potential to form tumours. When these cells are transplanted in the undifferentiated state, they form teratomas, tumours derived from all three germ layers. Currently, the only way to ensure that teratomas do not form is to differentiate the ES cells, enrich for the desired cell type, and screen for the presence of undifferentiated cells [3].

The first two of these problems could be avoided using dedifferentiation of somatic cells as a means to obtain autologous “patient specific” pluripotent stem cell lines. The capacity for dedifferentiation is retained in mammalian somatic cells, and the reprogramming technology has provided two strategies for the generation of pluripotent SCs from adult differentiated cells.

1. The denominated somatic cell nuclear transfer (SCNT) also called “therapeutic cloning” or nuclear cloning (NC): one hallmark in this field of research took place in 1996 with the birth of *Dolly*, the cloned sheep conceived by transfer of an adult differentiated cell nucleus to an enucleated unfertilized oocyte [4]. This groundbreaking discovery made evident that even somatic highly differentiated cells retain the intrinsic ability to revert to a zygote state and thus provide a potentially inexhaustible source of ES cells.
2. The generation of pluripotent cells from differentiated reprogrammed animal cells, known as induced pluripotent SCs, or IPS cells: one decade after the birth of *Dolly*, another decisive discovery brought the advent of IPS cells by transgenic expression of merely four transcription factors in adult somatic mouse cells, namely, *Oct3/4, Sox2, Klf4,* and *c-myc *[5]. This protocol also worked with adult human cells, using *OCT3/4, SOX2, KLF4,* and *c-MYC *[6] or *OCT-4, SOX-2, LIN28,* and *NANOG* [7].

Somatic cell nuclear transfer (SCNT) entails the removal of an oocyte nucleus followed by its replacement with a nucleus derived from an adult somatic cell [8]. SCNT has limitations; in addition to the serious ethical issues surrounding the cloning of human embryos created for research [9], the scarcity of fresh donated mature human metaphase-II oocytes of high quality available for research is a significant obstacle [10]. Currently, the efficiency of the overall cloning process is quite low as the majority of embryos derived from animal cloning do not survive after implantation [11]. At present, the medical applications of SCNT have been halted on account of the inefficacy of the process, the lack of knowledge of the underlying mechanism, and ethical concerns [12]. Nevertheless, nuclear transfer has shown that all genes required to create an entire organism are present in the nucleus of the differentiated cell and can be activated on exposure to reprogramming factors present in the oocyte [13]. In addition, SCNT is a powerful tool to probe the developmental potential of a cell, and the major conclusion from these findings was that development imposes reversible epigenetic rather than irreversible genetics changes on the genome during cellular differentiation [14]. However, generation of embryos directly from embryonic stem cells by tetraploid embryo complementation has become a popular means as an alternative to SCNT.

Somatic cells can be reprogrammed by fusion with ES cells, and Takahashi and Yamanaka concluded that ES cells contain factors that induce pluripotency, and these factors were also likely involved in the maintenance of pluripotency in ES cells. Based on this hypothesis, they showed that ectopic expression of defined transcription factors was sufficient to reprogram mouse embryonic fibroblasts and adult fibroblasts to pluripotent ES-like cells after retroviral-mediated transduction of the four transcription factors *Oct3/4*, *Sox2*, *c-myc,* and *Klf-4*, under ES culture conditions [5]. These cells, designated IPS cells, exhibit the morphology and growth properties of ES cells and express ES marker genes. Subcutaneous transplantation of IPS cells in nude mice resulted in teratomas. Injection of iPS cells into blastocyst contributes to chimaeras. IPS cells showed unlimited proliferation *in vitro* maintaining their pluripotency. Overexpression of the four factors generated cells capable of forming adult chimaeras and generating functional germ cells [15–18]. Human IPS cells, produced by expression of either* Oct-4, Sox-2*, *c-myc,* and *Klf-4 *or *Oct-4*, *Sox-2, Nanog,* and *Lin-28*, are remarkably similar to human ES cells [19]. However, controversy exists with regard to the differential gene expression profiles (genetic signatures) in ES and IPS cells [20, 21]. Consistent with this, IPS cells show attenuated potential differentiation in comparison to ES cells [22, 23].

A major limitation of reprogramming strategies is the use of potentially harmful genome integrating viruses to deliver reprogramming factor transgenes. Most IPS cells are prepared by viral vectors, such as retrovirus [5] and lentivirus [24], that integrate the reprogramming factors into host genomes, increasing the risk of tumor formation. The residual presence of integrated transgenes following the derivation of IPS cells is highly undesirable. The four factors used to induce reprogramming are strictly speaking oncogenes, thus implying a risk of transformation to a cancer phenotype. There are substantial grounds to state that the process of nuclear reprogramming by virus-assisted factor insertion in the cell genome increases the risk of carcinogenesis [25]. Supporting this, the efficiency of reprogramming increases largely in cells where the p53 tumor suppressor gene is knocked-out [26, 27]. This high risk of carcinogenesis is largely, but not exclusively, related to the integration of c*-MYC* transgenes [28, 29].

Alternative gene factor delivery systems include nonintegrating adenoviruses [30], plasmid transfection [31], doxycycline-inducible excisable *piggyBac *(PB) transposon system [32], and nonintegrating episomal vectors [33]. Another different strategy consists of delivery of recombinant proteins rather than genes into the cells to be reprogrammed [34, 35]. Others have explored the induction of reprogramming by chemical stimulation and screening/selection of effective small molecules, thus reducing the amount of factors delivered to cells [36]. Using the latter approach, there have been successful trials to generate IPS cells with the introduction of only one reprogramming factor (*OCT-4*) in multipotent neural SC [37] and dermal papilla cells from hair follicles [38]. The four factors that were initially identified can now be substituted with different factors or with certain small molecules, but the original finding—that a set of factors is required—holds true, and certain key gene factors such as *OCT-4* cannot be omitted.

The possibility to obtain patient-specific IPS cells has brought big hope on the prospect of future tissue engineering regenerative therapies by cell transplant since these new pluripotent cells circumvent two of the main problems traditionally associated with the sources of human pluripotent SCs: the serious ethical issues arising from the need to manipulate human embryos, and the possibility of rejection of the transplanted cells by the host immune system. However, the induction of carcinogenesis remains a pending threat. The potential of IPS cell technology is tremendous, but it will be essential to improve the methodologies for IPS cell generation and to precisely evaluate each clone and subclone of IPS cells for their safety and efficacy. It will be necessary to perform a detailed cellular and molecular study of somatic cells during their progression to pluripotent state [39]. Many aspects still remain to be clarified, given that the efficiency of the cell reprogramming process is certainly very low [40].

## 2. Tissue Engineering and IPS Cells: Definition and General Concepts

Tissue engineering aims to generate living tissues that could be used for the restoration of the function of several organs [41]. Tissue engineering has progressed from the use of biomaterials, which may repair or replace diseased or damaged tissue, to the use of controlled three-dimensional scaffolds in which cells can be seeded before implantation [42]. Organ shortage and suboptimal prosthetic or biological materials for repair or replacement of diseased or destroyed human organs and tissues are the main motivation for increasing research in the emerging field of tissue engineering in regenerative medicine [43–45]. While tissues such as bone or skin can effectively repair a small injury given sufficient time, many tissues such as myocardium, cartilage, and neural tissues do not regenerate properly without intervention [46].

**Figure 1:**
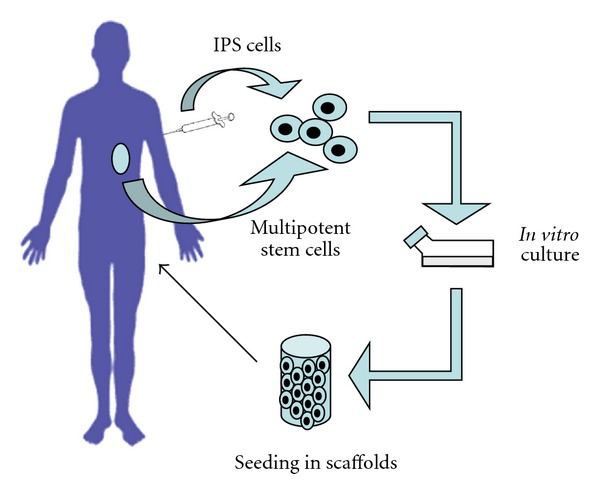
Basic scheme of tissue engineering. A biopsy is carried out to extract cells from the patient. These can be endogenous organ-specific multipotent SCs, or alternatively they can be adult differentiated somatic cells, reprogrammed to IPS cells. SCs are isolated, expanded and differentiated to the cell type of interest in an appropriate culture medium, seeded in a scaffold, and cultured *in vitro*. At this point, the new tissue is implanted in the patient.

The availability of sources of pluripotent SC has increased immensely the potential of cell therapy in medicine and opens up new perspectives in the treatment of diseases [47, 48]. IPS cells can proliferate and be induced to differentiate to a particular cell type, and the selected cells can be seeded in a specific mould or scaffold and cultured *in vitro*. Scaffolds (natural or synthetic) may be composed of polymers, metals, ceramics, or composites [49, 50]. Bioreactors are used to grow the cells on the scaffolds until the tissue or the organ is fully developed [41]. The cells can be expanded in culture and then reimplanted in the patient [51, 52] (Figure 1). The cells can come from the same individual (autologous) or the same species but from a different individual (allogeneic) or even can originate from different species (heterologous).

Nevertheless, because of the limitations inherent to the cell reprogramming process, it is advised that research on IPS cells in the field of tissue engineering is carried out in appropriate tissue and organ systems that permit a safe assessment of the long-term behavior of these reprogrammed cells. As we will discuss in the last part of the paper, the dental system may constitute a very good choice as a testing ground for IPS cells applied to tissue engineering, owing to several specific features of dental cells and tissues. We will proceed by briefly describing endogenous sources of dental SC, the process of normal tooth development and its associated structures, and finally we will discuss how these features may constitute a decisive advantage to investigate future applications of IPS cells in full dental regeneration.

## 3. Are Teeth the New Golden Mine of SC?

Teeth are nonvital organs that, remarkably, have proven to be a surprisingly rich source of multipotent ectomesenchymal SC (EMSC). The majority of live adult tooth tissues derive from the neural crest, and therefore all dental SCs considered here are collectively termed EMSCs. Teeth are easily amenable for extraction in the dental clinic, precluding the need of complex chirurgical care and invasive isolation methods. Owing to their amount and accessibility, dental tissues constitute one of the most consistent sources of human SCs that can be found nowadays. Human teeth are extracted and disposed of by thousands in dental clinics worldwide, the majority of them corresponding to third-molars (wisdom teeth) of young patients, which are usually removed for orthodontic reasons.

There are five different types of dental EMSCs that have been isolated and characterized: dental pulp SC, or DPSC [53], SC from human exfoliated deciduous teeth, or SHED [54], periodontal ligament SC, or PDLSC [55], SC from the apical papilla, or SCAP [56], and SC from dental follicle [57]. All these display stem cell features such as multilineage differentiation potential to various cell types including odontoblasts, cementoblasts, osteoblasts, chondroblasts, adipocytes, muscle cells, and neurons [58]. Notably, due to their neural crest origin, dental SCs are considered to be a good stem cell choice to generate neural and glial cell derivatives. Some of these cells, such as SHED, express early immature glial and neuronal cell markers in basal conditions, even in the absence of neurogenic stimulation [54].

Very recent research has described a population of *OCT-4+, NANOG+, LIN28+,* and *SOX-2+* cells in the dental pulp that is argued to constitute an endogenous dental source of pluripotent SCs [59]. These cells can be induced to differentiate to endoderm and mesoderm cell derivatives. If this finding got confirmed by other research teams in the near future, the field of dental stem cell research will no doubt receive a definite boosting. Other tested strategies to obtain pluripotent cells out of dental cells consist of reprogramming dental multipotent EMSC or adult gingival and periodontal fibroblasts to IPS. This has been successfully carried out by different research groups [60–62].

## 4. Potential of the Dental System for IPS Technology in Full Organ Bioengineering

Dental SCs have been successfully tested in tissue engineering research, where full generation of dentin pulp complexes and even whole teeth out of isolated cells (complete organ restoration) has proven to be possible [63]. The two cell types that take part in the generation of teeth come from different embryonic origins: surface epithelial (ectoderm) and ectomesenchymal (neural crest). Those tissues are precursors of the enamel organ and dental papilla, which will generate tooth enamel and the dentin-pulp complex, respectively. Tooth development takes place over different morphogenetic stages (placode, bud, cap, bell, appositional) and is governed through a complex series of epithelial-mesenchymal cellular inductions. As a consequence of continuous reciprocal signaling, the precursors of ameloblasts and odontoblasts, the two key mineralizing adult dental cell types, will elongate, polarize, and differentiate at the epithelium-mesenchyme interface. These ameloblastic (enamel producing) and odontoblastic (dentin producing) cells will terminally differentiate at the late bell-early appositional stage transition, and this will mark the beginning of secretion and deposition of hard enamel and dentin tissues, starting by the tooth cusps [64].

**Figure 2:**
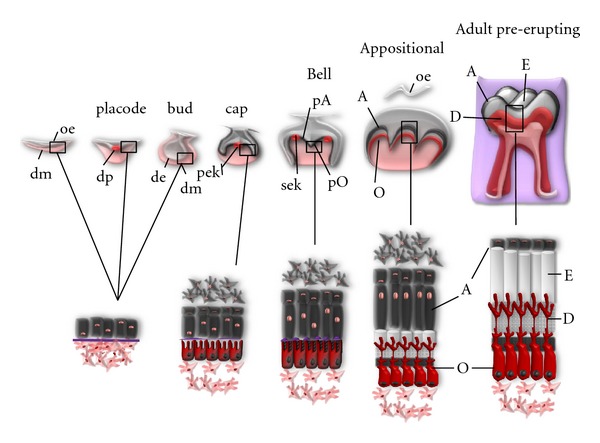
Stages and events of molar tooth development. Tooth morphogenesis is carried out by complex epithelium-ectomesenchyme interactions. Epithelial cells are depicted in gray and ectomesenchymal cells in red. As a consequence of sequential induction events, ameloblast (A) and odontoblast (O) cells start to differentiate at the interface between dental epithelium (de) and dental mesenchyme (dm) at the end of bell stage. Enamel (E) and dentin (D) tissues are secreted during the appositional stage, when the developing dental organ appears separated from the oral epithelium (oe). When enamel mineralization is completed, ameloblasts undergo regression, whereas odontoblasts will be maintained during the whole life of the tooth. The areas covered by squares are represented magnified below. Signaling centers during tooth morphogenesis are drawn as red circles: dental placode (dp), primary enamel knot (pek), and secondary enamel knot (sek). pA: preameloblasts; pO: preodontoblasts.

Importantly, once the deposition and maturation of tooth enamel is complete, ameloblastic cells will undergo a drastic regression, losing their elongated size and polarized state and mingling with adjacent epithelial cells to form the so-called “reduced enamel epithelium,” a transient coating structure that will end up disappearing at the moment of tooth eruption. The only epithelial cells that will remain in adult tooth structures are the epithelial cell rests of Malassez (ECRM), deriving from Hertwig's epithelial root sheath (HERS), another transient structure involved in dental root formation. ECRMs play no known role in the adult tooth and appear as little cell clusters in the periodontal ligament. On the contrary, ectomesenchyme-derived odontoblasts and dental pulp tissues will persist throughout the tooth life well into adulthood (Figure 2).

*De novo *generation of fully functional dentin-pulp complexes and periodontal tissues has been successfully accomplished by transplantation of endogenous EMSC to experimental animals, in combination with mineralized hydroxyapatite/tricalcium phosphate scaffold carriers [65]. Transplanted cells eventually assimilate and remodel the scaffold to create completely biological structures. Under these conditions, it is also possible to design a biological tooth root that supports the placement of a synthetic tooth crown, as shown by elegant studies [66]. With improvements and adaptations of this technique, in the future it may be possible to replace synthetic implants by biocompatible engineered tooth root tissues in humans. Therefore, endogenous EMSCs hold a big potential for their use in regenerative dentistry.

Even more striking was the reported generation of functional mouse teeth generated exclusively from dissociated dental SCs. In a sound study, Tsuji et al. [67] isolated dental epithelial cells from E14.5 cap stage mouse teeth and recombined them together with ectomesenchymal DPSC in a collagen gel, thus creating a bioengineered molar tooth germ. Remarkably, the bioengineered tooth proceeded normally through all the different morphogenetic stages and could be eventually transplanted into the jawbone of a host mouse, to create a fully functional adult tooth that integrated well into surrounding tissues, presented a correct occlusion, supported masticatory forces, could perform orthodontic movements, was normally innervated, and responded adequately to pain stimuli. This positive experience holds great promise in the field of full dental organ regeneration, which would no doubt revolutionize future dentistry.

**Figure 3:**
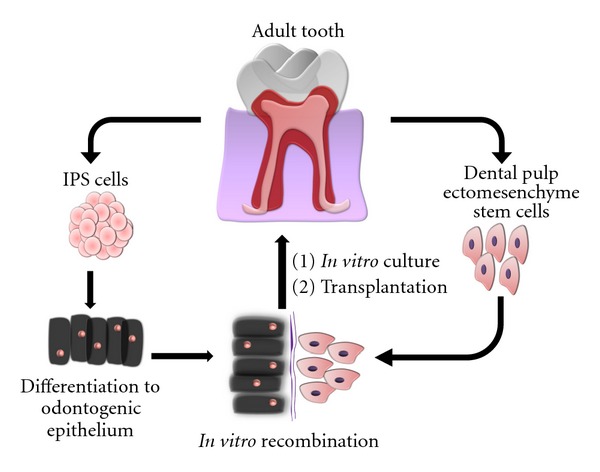
Theoretical design of a dental engineering process involving IPS cells. Tooth tissues already present well-characterized populations of ectomesenchymal SCs, that can generate *de novo* a complete dentin-pulp complex and periodontium. The hard enamel tissue constituting the remaining part of the tooth must be formed by dental epithelial cells. In this context, autogenic IPS cells could be used as a source of new dental epithelium, to be recombined with ectomesenchymal cells, thus creating a bioengineered tooth germ that can be cultured *in vitro* and transplanted to the jawbone/maxillary bone of a recipient host to form a fully functional tooth. Almost all IPS-derived epithelial cells will disappear after tooth eruption, as a consequence of normal dental development.

However, obviously much experimentation is required and major issues need yet to be solved before an approach like tooth germ engineering by dental stem cell recombination can be translated to the dental clinic. Probably the most important limiting factor is the absence of consistent sources of epithelial SC with odontogenic potential in the adult human individual, to be recombined with endogenous dental mesenchymal SCs. There has been substantial progress in the identification of possible epithelial substitutes, using PDL-derived ECRM [68], and postnatal oral mucosal epithelial cells [69]. Both these cell types can be cultured *in vitro* and induced to differentiate to ameloblastic cell lineages. Another realistic possibility, exclusively for research purposes, would constitute the rodent incisor, which contains an epithelial stem cell niche [70]. However, although the sources of endogenous dental epithelial SC seem to be scarce, an appealing alternative would be to obtain them from autogenic IPS cells, properly differentiated *in vitro*. Once this step is accomplished, the remaining process of recombination into collagen scaffold matrices, *in vitro* organ culture, and *in vivo* transplantation should not pose extreme technical difficulties. The final outcome would be a fully developed bioengineered human tooth obtained from dissociated autogenic EMSC and IPS cells, in which the latter would almost completely disappear after tooth eruption (Figure 3).

Thus, there are several arguments that point to teeth as a very attractive system to test IPS cells in a context of full organ restoration therapy.

- First, tooth development by epithelial (IPS derived) and ectomesenchymal (endogenous) autogenic or allogeneic cell recombination can be performed and followed up *in vitro* during early developmental stages up to two weeks, therefore permitting selection of the most appropriate or best-looking bioengineered teeth before transplant.
- Second, IPS cells in the context of tooth engineering are mostly needed as a source of dental epithelial cells and eventually enamel producing ameloblasts. These epithelial cell derivatives will be present only transiently and disappear after tooth eruption occurs, with the sole exception of ECRM. Therefore, the risk of IPS-induced tumorigenesis should be greatly reduced in the dental system.
- Third, it is possible to generate autogenic (patient-specific) IPS cells from dental cells and tissues to minimize the chance of immune rejection.
- Fourth, long-term outcomes of biological teeth can be readily followed during routine dental and periodontal check-ups, even by visual exploration.
- Finally, should complications arise because of the use of SC or IPS cells (tumorigenic or other), extraction of the tooth piece can be performed with relative simplicity, by noninvasive procedures and with no life-threatening risk to the patient.

At the present time, we have no means to predict what will be the future of IPS technology to treat human diseases by cell therapy, but the recently discovered process of adult cell reprogramming still continues to fascinate the research community. No doubt that development of safe nontumorigenic IPS cell lines will have a great deal to do with their eventual success [71, 72]. Time will tell whether teeth will become an important testing ground for applications of IPS cells in a complex context of tissue regeneration, with multiple cells of different lineages, IPS and host, that will need to coordinate and communicate with each other. It seems clear that we will learn a lot about IPS cell integration on tissues over the next decades.
